# Counteracting the Common Shwachman–Diamond Syndrome-Causing *SBDS* c.258+2T>C Mutation by RNA Therapeutics and Base/Prime Editing

**DOI:** 10.3390/ijms24044024

**Published:** 2023-02-16

**Authors:** Laura Peretto, Elena Tonetto, Iva Maestri, Valentino Bezzerri, Roberto Valli, Marco Cipolli, Mirko Pinotti, Dario Balestra

**Affiliations:** 1 Department of Life Sciences and Biotechnology and LTTA, University of Ferrara, 44121 Ferrara, Italy; 2 Department of Translational Medicine and for Romagna, University of Ferrara, 44123 Ferrara, Italy; 3 Cystic Fibrosis Center, Azienda Ospedaliera Universitaria Integrata di Verona, 37126 Verona, Italy; 4 Department of Medicine and Surgery, University of Insubria, 21100 Varese, Italy

**Keywords:** *SBDS*, Shwachman–Diamond syndrome, U1snRNA, base editor, prime editor, genome editing, trans-splicing, PTM

## Abstract

Shwachman–Diamond syndrome (SDS) represents one of the most common inherited bone marrow failure syndromes and is mainly caused by *SBDS* gene mutations. Only supportive treatments are available, with hematopoietic cell transplantation required when marrow failure occurs. Among all causative mutations, the *SBDS* c.258+2T>C variant at the 5′ splice site (ss) of exon 2 is one of the most frequent. Here, we investigated the molecular mechanisms underlying aberrant *SBDS* splicing and showed that *SBDS* exon 2 is dense in splicing regulatory elements and cryptic splice sites, complicating proper 5′ss selection. Studies ex vivo and in vitro demonstrated that the mutation alters splicing, but it is also compatible with tiny amounts of correct transcripts, which would explain the survival of SDS patients. Moreover, for the first time for SDS, we explored a panel of correction approaches at the RNA and DNA levels and provided experimental evidence that the mutation effect can be partially counteracted by engineered U1snRNA, trans-splicing, and base/prime editors, ultimately leading to correctly spliced transcripts (from barely detectable to 2.5–5.5%). Among them, we propose DNA editors that, by stably reverting the mutation and potentially conferring positive selection to bone-marrow cells, could lead to the development of an innovative SDS therapy.

## 1. Introduction

Shwachman–Diamond syndrome (SDS; Online Mendelian Inheritance in Man [OMIM] database entry#260400) is a rare recessive autosomal disease characterized by multiple organ involvement, including exocrine pancreatic insufficiency, impaired hematopoiesis, leukemia predisposition, and bone malformations. Patients with SDS have a high risk of myelodysplastic syndrome (MDS) and acute myeloid leukemia (AML), which are currently considered the major causes of mortality [1]. SDS is caused by mutations in the Shwachman-Bodian-Diamond Syndrome (*SBDS*) gene, which encodes the homonymous protein SBDS, which is mainly involved in ribogenesis. To date, only supportive treatments are available, and hematopoietic cell transplantation represents the sole therapy for marrow failure, which poses the need for novel therapeutic strategies. 

Among the over 20 different *SBDS* mutations so far identified, the c.183-184TA>CT and c.258+2T>C mutations, originally reported as 183-184TA>CT and 258+2T>C, are the most common [2]. These recurring mutations often arise from gene conversion events with the pseudogene copy (*SBDSP*) which shares 97% nucleotide homology with *SBDS*. While the c.183-184TA>CT change results in the creation of a premature termination codon (K62X), the c.258+2T>C variant at the 5′ splice site (ss) is associated with aberrant pre-mRNA splicing due to the usage of an upstream cryptic 5′ss at positions c.251-252 [2], eventually resulting in an 8-bp deletion and frameshift (84Cfs3). Importantly, the c.258+2T>C pathogenic variant has been found in more than 90% of worldwide SDS patients [2,3]. These features make the c.258+2T>C mutation an attractive target for correction approaches acting on pre-mRNA splicing, which would preserve the natural gene regulation only in physiological tissues.

Among RNA therapeutics acting on pre-mRNA splicing, modified U1snRNA and antisense oligonucleotides (AON) represent some of the most attractive, with numerous experimental evidence in cellular and animal models [4,5,6,7,8,9,10] and also in humans [11,12]. In particular, either the compensatory or the exon-specific (ExSpe) U1 variants of U1snRNA, the RNA component of the spliceosomal U1 ribonucleoprotein (U1snRNP) mediating 5′ss recognition in the earliest splicing steps [13], were shown to rescue splicing mutations both in cellular and animal models of human diseases [14,15,16,17,18,19]. Moreover, triggering splicing between different RNA molecules (trans-splicing) might represent an additional correction option, albeit suffering from an intrinsic low efficiency [20,21]. On the other hand, DNA editing would offer a life-long cure with a single intervention, also from the perspective of ex vivo gene therapy for hematopoietic bone marrow cells. Intriguingly, the recently developed base and prime editors (BE and PE, respectively) represent novel approaches to expand the scope of donor-free precise DNA editing without requiring any double-strand breaks [22,23,24]. They represent the latest CRISPR genome-engineering toolkits constituted by engineered proteins where a Cas9 nickase is fused with a base modification (deaminase) or reverse transcriptase enzyme, respectively [25,26]. While the BE is able to efficiently mediate all four possible transition mutations (C→T, A→G, T→C, and G→A), the PE can revert all types of point mutations, small insertions, or deletions in a precise and targeted manner. Overall, DNA base- and prime-editing tools enable precise and efficient nucleotide substitutions in a programmable manner, without requiring a donor template, thus opening the possibility to rescue the majority of pathogenic mutations [25,26]. 

Here, we investigated the molecular mechanisms underlying aberrant *SBDS* splicing triggered by the common c.258+2T>C mutation and explored splicing-switching strategies based on engineered U1snRNAs or trans-splicing molecules and BE and PE DNA editing to revert the mutation at the DNA level; the study of which led us to propose DNA editing as the most promising approach for future development.

## 2. Results

### 2.1. The SBDS c.258+2T>C Mutation Is Associated with Trace Levels of Correctly Spliced Transcripts

**Figure 1:**
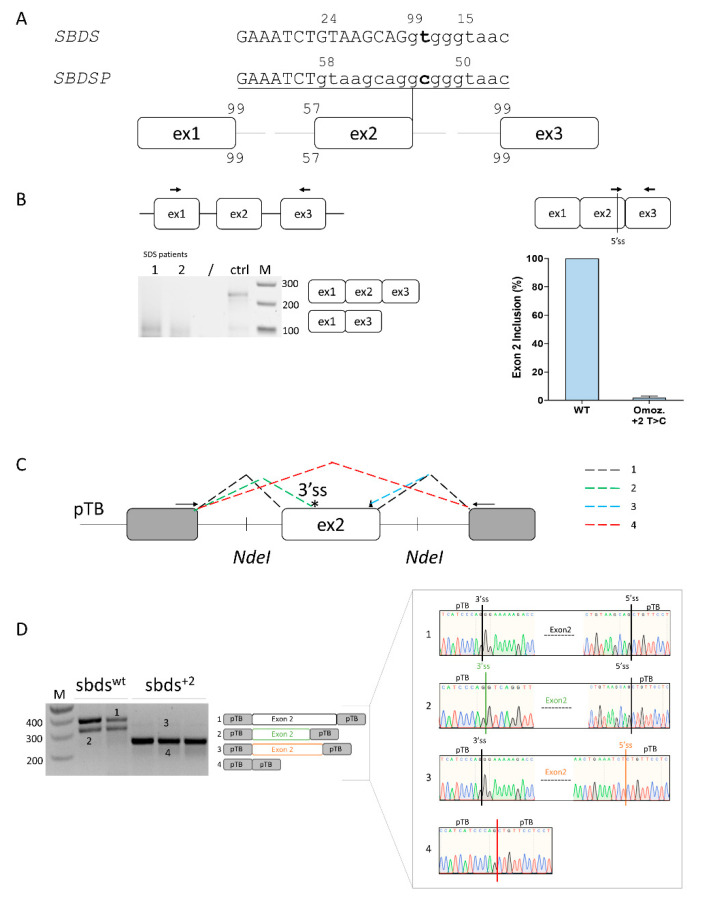
Ex vivo and in vitro evaluation of *SBDS* exon 2 inclusion. (**A**) Schematic representation of the *SBDS* and *SBDSP* pre-mRNAs focused on exon 2, with exons and introns indicated by boxes and lines, respectively. The numbers indicate the score of 3′ss and 5′ss in both *SBDS* and *SBDSP* contexts. Intron and exon sequences are in lower and upper case, respectively; (**B**) Evaluation of *SBDS* transcripts (left) and quantification of *SBDS* exon 2 inclusion (right) in immortalized lymphoblastoid cells derived from SDS patients homozygous for the c.258+2T>C mutation or normal subject as a control. The schematic representation of the *SBDS* mRNA context, with primers exploited to evaluate splicing patterns and quantify correctly spliced mRNA, is indicated above. Amplified products were separated on 2.5% agarose gel. M, 100 bp molecular weight marker; (**C**) Schematic representation of the minigene, containing the human *SBDS* exon 2 genomic sequence. The fragment was cloned as in the pTB vector by exploiting the *NdeI* sites. Exonic and intronic sequences are represented by boxes and lines, respectively. The colored dotted lines recapitulate the alternative usage of the splice sites, which originate the different transcript isoforms (1 to 4) detected by in vitro splicing analysis and reported in panel D. Primers (arrows) used to perform the RT-PCR are indicated on top. The 3′ and 5′crypric sites are indicated with an asterisk and a triangle, respectively; and (**D**) Evaluation of *SBDS* splicing patterns in HEK293T cells transiently transfected with wild-type (sbds^wt^) or mutated (sbds^+2^) minigenes. The schematic representation of the transcripts (with exons not in scale) is reported in the middle. Numbers represent correctly spliced transcripts (1), those arising from the usage of the cryptic 3′ss (2), of exonic 5′ss (−8 bp) (3), or from exon 2 skipping (4), respectively. Amplified products were separated on 2.5% agarose gel. M, 100 bp molecular weight marker. Sanger sequencing of transcripts is reported on the right.

Computational analysis predicts that *SBDS* exon 2 is poorly defined, with a strong 5′ss (score: 99), a weak 3′ss (57), and the presence of alternative 5′ss located upstream (−8 bp, score 24) and downstream (+5, score 15) of the authentic one, together with an intronic cryptic 3′ss (score 77) located at +58bp. The c.258+2T>C change would virtually abrogate the authentic 5′ss and strengthen the surrounding cryptic 5′ss (Figure 1A).

The *SBDS* exon 2 inclusion efficiency was evaluated in lymphoblastoid cell lines from a c.258+2T>C homozygote and a normal subject as a control. Splicing patterns in the control cells revealed, besides correctly spliced transcripts, trace levels of transcripts lacking *SBDS* exon 2. On the other hand, patterns in immortalized lymphoblastoid cell lines derived from an SDS patient indicated that the mutation leads to exon 2 skipping, but when assessed by qPCR, it is also compatible with trace levels of correct transcripts (2%) (Figure 1B). 

To dissect the aberrant splicing mechanisms, we created an exon trapping minigene including *SBDS* exon 2 and its flanking intronic sequences (Figure 1C), and transiently expressed it in HEK293T cells. Splicing patterns evaluated by RT-PCR with transgene-specific primers revealed that the wild-type minigene (sbds^wt^) also undergoes alternative splicing through the usage of a cryptic 3′ss at positions c.186-187 (band 2) (Figure 1D). On the other hand, the introduction of the c.258+2T>C mutation (sbds^+2^) triggers exon 2 skipping (band 3). To better evaluate the presence of poorly represented transcripts, we exploited fluorescent labeling of RT-PCR products followed by denaturing capillary electrophoreses (Figure S1) that revealed the usage of the proximal exonic cryptic 5′ss and, upon overloading of the column, also trace levels of correctly spliced forms, roughly accounting for 0.2% of all transcripts. 

Altogether, these ex vivo and in vitro data support the association of the mutation with trace amounts of correct transcripts, and thus residual SBDS protein expression.

### 2.2. SBDS Exon 2 Is Dense in Splicing Regulatory Elements and a Compensatory U1snRNA Can Force Proper Exon Definition

**Figure 2:**
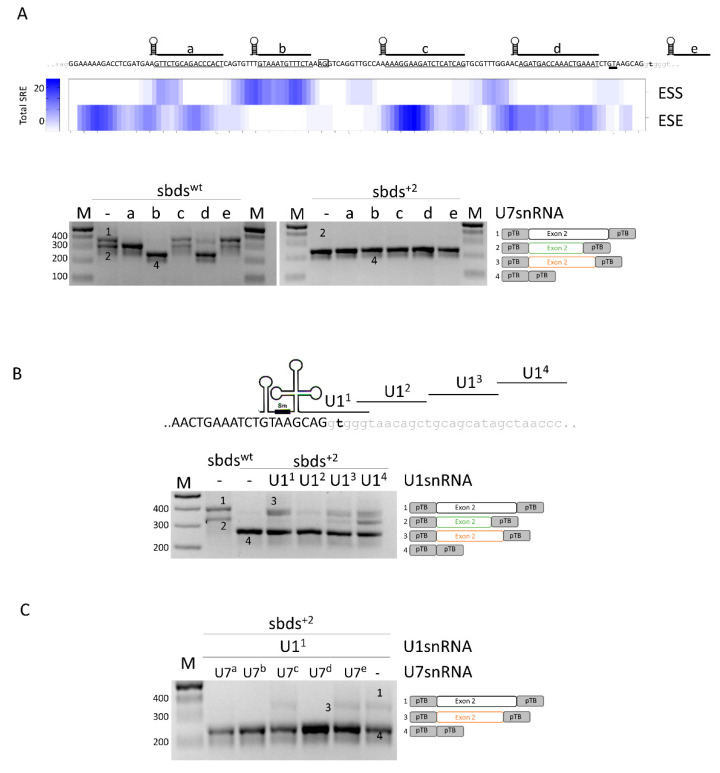
Analysis of splicing regulatory elements (SRE) of *SBDS* exon 2 and rescue of c.258+2T>C mutation with engineered U1snRNAs. (**A**) Upper panel: sequences of *SBDS* exon 2 and surrounding introns are indicated in upper and lower case, respectively. The regions masked by the engineered U7snRNAs, indicated above the sequence, are underlined. The highly conserved dinucleotides of the exonic cryptic 3′ss and 5′ss are boxed and double underlined, respectively. The total number of exonic splicing enhancers (ESE) and exonic splicing silencers (ESS) is reported in the heat map. SRE: Splicing Regulatory Elements. Lower panel: Evaluation of *SBDS* splicing patterns in HEK293T cells co-transfected with sbds^wt^ or sbds^+2^ minigenes alone or with engineered U7snRNAs. The schematic representation of transcripts is reported on the right; (**B**) Schematic representation of *SBDS* exon 2, in upper case, intron 2, in lower case, and of U1snRNA variants exploited to redirect the spliceosome to the mutated 5′ss. The splicing pattern of HEK293T cells transfected with wild-type or mutated minigenes alone or in combination with U1snRNAs is indicated below. The schematic representation of transcripts is reported on the right; and (**C**) Evaluation of *SBDS* splicing patterns in HEK293T cells co-transfected with the sbds^+2^ minigene, U1^1^snRNA, and U7snRNA variants. Amplified products were separated on 2.5% agarose gel. M, 100 bp molecular weight marker. The schematic representation of the transcripts (with exons not in scale) is reported on the right.

To help design splicing-switching molecules for correction attempts, we sought exonic splicing regulatory elements which were bioinformatically predicted within *SBDS* exon 2. To evaluate their functional role, we expressed variants of the U7snRNA (U7a to U7e) designed to base-pair and mask most of the regulatory elements (Figure 2A, upper panel). In co-transfection experiments with the sbds^wt^ minigene, the U7c did not exert any effect, while the U7b and U7d resulted in exon 2 skipping. U7a and U7e, instead, were associated with alternatively spliced exon 2 transcripts resulting from the usage of the cryptic 3′ss (Figure 2A, lower panel; Figure S1) and slightly increased in correctly spliced transcripts. Differently, co-transfection of engineered U7snRNAs with the sbds^+2^ mutant minigene did not show any effect, being the exon 2 skipping the main effect. 

In the attempt to force proper exon 2 definition, we designed a panel of engineered U1snRNA variants, either compensatory (U1^1^) or exon specific (U1^2,3,4^) (Figure 2B). In co-transfection experiments with engineered U1snRNA, analysis of splicing by denaturing capillary electrophoreses revealed that all U1snRNAs strongly promoted the usage of the proximal cryptic exonic 5′ss. Moreover, the exon-specific U1^3^ and U1^4^ also promoted the usage of the exonic cryptic 3′ss. The complementary U1^1^ led also to a slight increase in the relative proportion of (2.5 ± 0.8%) correctly spliced transcripts (Figure S1). 

Altogether, these data do not support a correction approach based on antisense molecules but selected a compensatory U1snRNA that, albeit with low efficiency, promotes proper exon 2 definition and the synthesis of correct transcripts. 

### 2.3. A 3′ Trans-Splicing Molecule Can Mediate Trans-Splicing of SBDS Exon 2

**Figure 3:**
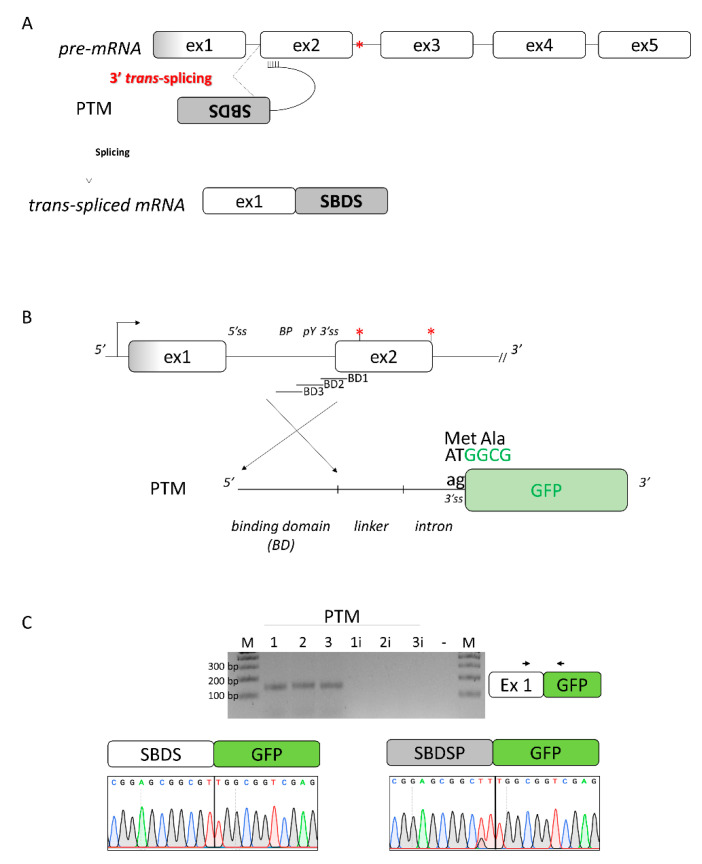
Design of the 3′ pre-trans-splicing molecule (PTM) for *SBDS* exon 2 and trans-splicing studies. (**A**) Schematic of the trans-splicing process. The complete *SBDS* and *SBDSP* transcript region from exon 2 to 5, with the c.258+2T>C mutation indicated by the red asterisk, is replaced by its wild-type copy provided by the PTM. Exons and introns are not in scale; (**B**) Schematic representation of the PTMs designed to induce *SBDS* trans-splicing. The c.258+2T>C and c.183-184TA>CT mutations are indicated by red asterisks. The sequence of the green fluorescence protein (GFP), without the initial AT dinucleotide of the first triplet and exploited to evaluate the occurrence of the trans-splicing process, is reported in green. BD: binding domain of the PTM molecules; and (**C**) Evaluation of trans-splicing in HEK293T cells co-transfected with PTM or PTMi, exploited as controls. The position of primers is indicated by arrows, and the expected transcript is indicated on the right. Amplified products were separated on 2.5% agarose gel. M, 100 bp molecular weight marker. Sanger sequencing of transcripts is reported on the bottom.

Since both *SBDS* and its pseudogene *SBDSP* are expressed within cells, and they share high homology (Figure S2), we envisioned creating a 3′ pre-trans-splicing molecule (PTM) to explore a dual-target trans-splicing correction approach (Figure 3A). The PTM consisted of the 3′ region of *SBDS* intron 1, inserted in the reverse-antisense orientation, a linker region, the 3′ portion of a well-defined intron (from the alpha-globin gene), and the GFP expression cassette (Figure 3B). Three PTM molecules (PTM1 to 3), differing in the Binding Domain (BD) of the included 3′region of the intron, were generated. The generation of the corresponding PTM (PTM1i to 3i) with the intronic region cloned in the forward orientation represented negative controls. To easily identify trans-spliced transcripts, we exploited the GFP reporter gene instead of the *SBDS* coding sequence. To evaluate the occurrence of trans-splicing, the 3′ PTM variants as well as the PTM controls were transfected in HEK293T cells where *SBDS* expression was previously demonstrated (Figure S3). Analysis of GFP expression by FACS analysis did not show an appreciable increase in fluorescence in cells expressing the PTM as compared to controls (data not shown). The presence of trans-spliced transcripts was then investigated by a specific RT-PCR, followed by a nested PCR (Figure 3C), which ensured high sensitivity. The TA cloning of amplicons followed by the sequencing of transcripts revealed the presence of trans-spliced transcripts, with the GFP mRNA fused with *SBDS* and *SBDSP* exon 1, the latter harboring two amino acid changes (p.G24R and p.V43L) compared to the former. The high homology sequence (98%) at the DNA level of *SBDS* and *SBDSP* exon 1, and the impossibility of designing a trans-spliced positive control prevented the quantification of trans-spliced transcripts by specific qPCR.

Overall, these data provided experimental evidence that a 3′PTM can induce trans-splicing with both the *SBDS* and *SBDSP* pre-mRNA. 

### 2.4. The SBDS c.258+2T>C Variant Can Be Rescued by BE and PE Editors

**Figure 4:**
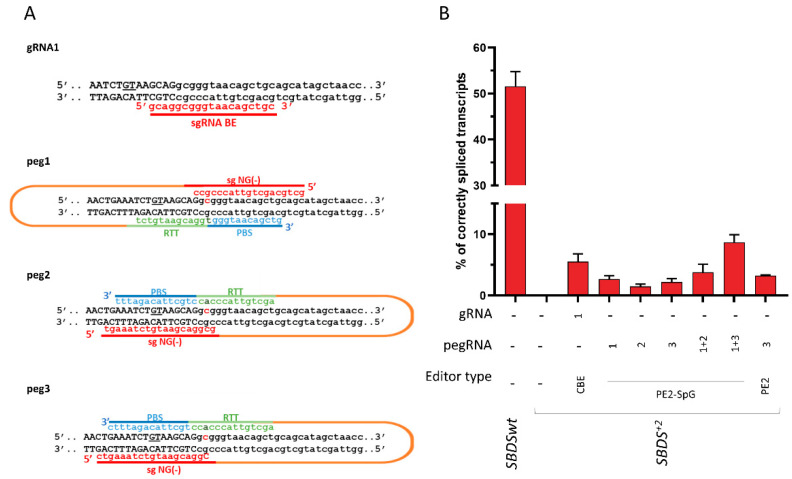
Development of base (BE) and prime (PE) editors to correct the *SBDS* c.258+2T>C mutation. (**A**) Schematic of the designed BE and PE editors. The sequence of the sense and antisense DNA strands, with exon and intronic sequences in upper and lower case respectively, is reported. The cryptic 5′ss located 8 bp upstream of the *SBDS* exon 2 5′ss is underlined. The c.258+2T>C mutation, on both DNA strands, is in bold. The spacer RNA, and the PBS, RTT, and gRNA scaffold sequences are indicated in red, blue, green, and orange, respectively. (**B**) Evaluation of correctly spliced transcripts through the denaturing capillary electrophoresis of labeled mRNA transcripts. Histograms report the amount of correctly spliced transcripts. Results are presented as the mean ± SD of three independent experiments. CBE: evoAPEBEC1-BE4mac-NG (Addgene plasmid no. 125616), PE2-SpG (Addgene plasmid no. 159978), and PE2 (Addgene plasmid no. 132775).

To evaluate the ability of the BE and PE editors to revert the c.258+2T>C mutation, and thus its detrimental effect on splicing, different gRNA and pegRNA was designed. In particular, one gRNA with the targeting nucleotide located at position +6 and 5 pegRNA targeting both NGG and NG PAM were constructed. The plasmids coding for BE or PE (g/pegRNA plus coding-editor plasmids) were transiently co-transfected with the *SBDS* splicing trapping plasmid into HEK293T cells. Moreover, the dual peg strategy was exploited [27,28]. The ability of the BE and PE to revert the causative mutation and thus restore proper mRNA processing was investigated by the fluorescent labeling of amplicons followed by denaturing capillary electrophoresis (Figure 4 and Figure S4). 

Analysis of the splicing pattern showed that treatment with the base editor resulted in correctly spliced transcripts, which accounted for 5.5 ± 1.3% of all transcripts. On the other hand, all PEs were able to rescue splicing, with corrections ranging from 1.5 ± 0.4% (PE2-SpG with peg2) to 3.3 ± 0.5% (PE2 with peg3). Interestingly, the correction observed with the dual PE strategy was higher than that observed with the single PE approach (from 2.7 ± 0.5%, 1.5 ± 0.4%, and 2.2 ± 0.6% to 3.8 ± 1.3% and 8.7 ± 1.2%).

Overall, the BE and PE approaches can be exploited to rescue the common *SBDS* c.258+2T>C variant leading to proper *SBDS* pre-mRNA splicing.

## 3. Discussion

Due to its occurrence at the highly conserved 5′ss GT dinucleotide, the SDS-causing *SBDS* c.258+2T>C mutation is commonly considered a null mutation. However, the identification of homozygous c.258+2T>C patients and the early embryonic lethality observed in *SBDS* knockout mice [29] suggest that residual SBDS expression is essential for survival. For this reason, we investigated the presence of *SBDS* correct transcripts in an EBV-transformed B lymphoblastoid cell line from a homozygous patient. This investigation, and particularly the exploitation of qPCR, enabled us to demonstrate the presence of a tiny amount of correctly spliced transcripts (~2%), a finding explaining the survival of patients homozygous for this mutation. In accordance, a residual extent of correct splicing was further demonstrated by expression of the *SBDS* mutant minigenes, which also revealed, besides the exon 2 skipping event, the usage of a cryptic splice site which was not appreciable ex vivo likely because of the nonsense-mediated decay effect. Altogether, these data on the residual usage of the correct 5′ss, which has key pathophysiological implications for SDS, are in agreement with recent research showing that GT>GC variants at 5’ss can be compatible with correctly processed mRNA [30,31,32,33].

Prompted by the minigene expression system, we dissected the splicing profiles of the affected *SBDS* affected region and experimentally demonstrated that exon 2 is not well defined, with several splicing regulatory elements that have been tested with a panel of engineered U7snRNAs designed to mask bioinformatically predicted splicing elements. As a matter of fact, U7a mainly promoted the usage of the cryptic 3′ss to indicate an underlying exonic splicing enhancer (ESS) contributing to the recognition of the authentic 3′ss. Concomitantly, the U7b and U7d mainly resulted in exon 2 skipping, suggesting the presence of additional ESS for proper exon 2 definition. It is worth noting that all effective U7 variants also promoted the usage of the cryptic exonic 5′ss, indicating a series of ESS guaranteeing the precise exon 2 5′ss definition. Intriguingly, the effect of the U7e pointed toward an increased exon 2 inclusion and thus a candidate intronic splicing silencer. Intrigued by the possibility to force proper exon 2 inclusion by acting on silencers, the entire panel of U7 was challenged in the mutated minigene but none resulted in an appreciable rescue. We, therefore, explored RNA therapeutics acting on pre-mRNA splicing based on modified U1snRNAs that were proven to be effective in rescuing splicing in numerous cellular and animal models [16,17,34,35,36,37]. The screening via splicing assays of a panel of engineered U1snRNAs base-pairing with the mutated 5′ss (compensatory), or targeting downstream intronic sequences (exon-specific U1; ExSpeU1), selected a compensatory one that partially restored usage of the mutated 5′ss, with correctly spliced transcripts increasing from barely detectable to 2.5% of the total transcripts. On the other hand, all engineered U1snRNAs promoted the usage of the cryptic, exonic 5′ss, thus suggesting that they favored the recruitment of the spliceosome and increased exon 2 inclusion, which vanished due to the competition of the strong cryptic splice site. Altogether, these data further highlight the complexity of proper exon definition and the interplay between several cis and trans-acting elements. 

Due to the difficulty of directly rescuing pre-mRNA splicing and intrigued by the possibility of targeting both the highly homologous *SBDS* and *SBDSP* pre-mRNAs, we explored spliceosome-mediated pre-mRNA trans-splicing (SmaRT). So far, the most effective reported SmaRT approaches have been applied in the replacement of 3′ pre-mRNA termini [38,39,40,41,42,43] in an attempt to replace most of the affected gene and thus expand the therapeutic potential. Based on the notion that almost all *SBDS* mutations (~80%) reported in SDS patients occur in exon 2, a 3′-PTM molecule was designed to target intron 1 for the future aim of replacing the region downstream of exon 2. For our pioneer attempt, the 3′PTM was equipped with the defective GFP coding sequence that enables a fluorescence-based detection as well as the discrimination of the trans-spliced transcripts from the HEK293T endogenous ones. While we did not observe any appreciable increase in fluorescence, the occurrence of SmaRT and therefore trans-spliced transcripts was demonstrated by RT-PCR with all 3′-PTM molecules. Intriguingly, sequencing indicated that SmaRT occurred both in *SBDS* and *SBDSP,* but the high sequence homology precluded the estimation of the relative proportion between the two SmaRT products. It is worth noting that the *SBDSP* exon 1 possesses only two amino acid changes (p.G24R and p.V43L) compared to the *SBDS* counterpart (Figure S5), which might be compatible, particularly the p.V43L change, with a functional SBDS protein variant. Our findings encourage studies on the characterization of this variant which would provide a strong rationale for further development of SmaRT-based dual pre-mRNA targeting. 

Through the exploitation of the mutated minigene expression system, we, therefore, tested a panel of cytosine base as well as prime editors and demonstrated that all of them corrected the mutation, as witnessed by the appearance of correctly spliced transcripts. More specifically, and in accordance with previous studies [27,28], the twin PE approach, where two PE editors code for complementary and edited DNA strands, was more efficient than the single PE editor, with the resulting proportion of correct transcripts reaching over 5% of all forms.

## 4. Materials and Methods

### 4.1. Creation of Recombinant Plasmids

To create the *SBDS* minigene, the genomic region of the human *SBDS* gene (LRG_104; NM_016038.4) including the last 578 bp of intron 1, exon 2 (130 bp), and the first 442 bp of intron 2 was amplified from the genomic DNA of a healthy subject using high-fidelity Q5 DNA-Polymerase (ThermoFisher Scientific, Waltham, MA, USA) and primers SBDS IVS1 NdeI F and SBDS IVS2 NdeI R, and subsequently cloned into the pTB plasmid by exploiting the *NdeI* restriction site inserted within primers. The c.258+2T>C variant was introduced by site-directed mutagenesis. Expression vectors for the U1snRNA and U7snRNA variants were created as previously reported [6]. Plasmids expressing sgRNAs and pegRNAs were constructed by the ligation of annealed oligonucleotides into the *BsmBI*-digested acceptor vector (Addgene plasmid no. 65777) and *BsaI*-digested acceptor vector (Addgene plasmid no. 132777), respectively. 

To create the pre-mRNA trans-splicing molecules (PTMs), the coding sequence of EGFP (without the first two nucleotides of the starting codon) was fused with the optimized 3′ portion of IVS1 of the HBB gene. The binding domain (BD), cloned in the correct or opposite orientation as the experimental control, was inserted between the *HindIII* and *BamHI* restriction sites. 

All plasmids were validated by Sanger sequencing. All primer sequences are listed in Table S1. 

### 4.2. Expression in Mammalian Cells and mRNA Studies

HEK293T cells were cultured and seeded in 12-well plates and transiently transfected with 1 µg of each expression vector using Lipofectamine 2000 reagent (ThermoFisher Scientific, Waltham, MA, USA), as previously described [44]. Twenty-four hours post-transfection the total RNA was isolated with Trizol (ThermoFisher Scientific, Waltham, MA, USA), reverse transcribed with random primers with RT-MMLV (Thermo Fisher Scientific, Waltham, MA, USA), and amplified with primers Alpha2,3 globin F, and Bra2 Rev oligonucleotides designed on the upstream and downstream exons, respectively. The PCR was run for 40 cycles at the following conditions: 30 s at 95 °C, 30 s at 56 °C, and 50 s at 72 °C, and amplicons were resolved on 2.5% agarose gel. For denaturing capillary electrophoresis analysis, the fragments were fluorescently labeled by using primer Bra2 Rev labeled with FAM and run on an ABI-3100 instrument (ThermoFisher Scientific, Waltham, MA, USA), followed by the analysis of peaks. All data reported are expressed as the mean ± standard deviation (SD) and derived from at least three independent experiments.

To evaluate the trans-splicing between the PTM molecule and the endogenous *SBDS* transcript, HEK293T cells were transiently co-transfected with 1 µg of PTM-expressing plasmid. Forty-eight hours post-transfection, total RNA was isolated, reverse transcribed with random primers, and amplified with primers SBDS 1F and EGFP R oligonucleotides.

### 4.3. Studies in Immortalized Lymphoblastoid Cell Lines

Wild-type and homozygous c.258+2T>C EBV-transformed B lymphoblastoid cell lines were cultured with RPMI-1640 (Sigma-Aldrich, St. Louis, MO, USA) medium supplemented with 10% fetal bovine serum (FBS) (Sigma, St. Louis, MO, USA), 1% L-glutamine, and 1% penicillin/streptomycin at 37 °C in a humidified atmosphere with 5% CO_2_. Total RNA, extracted with Trizol (Life Technologies, Carlsbad, CA, USA), was reverse-transcribed with random primers with RT-MMLV (Thermo Fisher Scientific, Waltham, MA, USA). The presence of correctly spliced *SBDS* transcripts was explored by quantitative PCR (qRT-PCR) with SsoAdvanced Universal SYBER Green Supermix (Bio-Rad, Hercules, CA, USA) according to the supplier’s protocol on a CFX connect qPCR system (Bio-Rad, Hercules, CA, USA) with primers qSBDS ex2F and qSBDS ex3R (Supplementary Table S1). Human glyceraldehyde-3-phosphate dehydrogenase (GAPDH) was exploited as a housekeeping gene. Each sample was run in triplicate. Cq and melting curves were acquired by use of Bio-Rad CFX Manager 3.1 software (Bio-Rad, Hercules, CA, USA). The mRNA levels were expressed as the relative expression index of 2-ΔΔCt. Values were expressed as the mean fold change ± standard error of the mean.

### 4.4. Computational Analysis

The computational prediction of splice sites was conducted by using the SliceRover (http://bioit2.irc.ugent.be/rover/splicerover, accessed on 15 March 2021) tool. The HOT-SKIP tool (https://hot-skip.img.cas.cz/, accessed on 15 March 2021) was used to predict the presence of exonic splicing regulatory motifs.

## 5. Conclusions

The strong unmet medical need for Shwachman–Diamond syndrome (SDS), one of the most common inherited bone marrow failure syndromes with only supportive treatments available and hematopoietic cell transplantation needed for the frequent marrow failure (88–98% of patients), pretenses the quest for intense research on alternative therapies. Here, for the first time, we explored a panel of correction strategies acting at the mRNA and DNA level and focused on a very common SDS-causing mutation, the *SBDS* c.258+2T>C splicing variant. Overall, these data indicated that the mutation is compatible with a tiny number of correct transcripts, which would account for residual SBDS protein expression and explain the survival of SDS patients. These minimal levels of correct transcripts can be increased by forcing the definition of the defective exon 2 by a compensatory U1snRNA variant or by trans-splicing, which also targets the highly homologous *SBDS* pseudogene’s pre-mRNA, with a potential additive effect on the SBDS protein expression and therapeutic impact. However, the relatively modest effects of these RNA-based correction approaches together with the required long-lasting expression of the therapeutic U1snRNA/PTM molecules, crucial for highly dividing cells such as bone marrow, weaken the interest in their further optimization and development. On the other hand, by targeting the c.258+2T>C mutation at the DNA level through cytosine base or prime editors, the correct nucleotide appeared to be installed, thus resulting in an appreciable increase in correctly spliced transcripts reaching over 5% in all forms. This represents the first proof-of-principle of the BE and PE-mediated correction of the c.258+2T>C mutation that would be permanent, transmitted to the daughter’s cells in proliferating tissues, and maintaining the physiological *SBDS* gene regulation. These data encourage further research aimed at optimizing this approach by exploring viral and non-viral delivery as well as novel generations of base editors, combined with the careful evaluation of target specificity. This could eventually lead to an “SDS personalized therapy” based on the autologous transplantation of edited hematopoietic stem cells. 

## 6. Patents

M.P. is an inventor in the patent (PCT/IB2011/054573) on modified U1snRNAs. All other authors declare no competing interests.

## Data Availability

All data generated or analyzed during this study are included in this published article and its supplementary information files.

## Supplementary Materials

The following supporting information can be downloaded at: https://www.mdpi.com/article/10.3390/ijms24044024/s1, List of primers used in the study [45].
