# Supplementary material for: Counteracting the Common Shwachman–Diamond Syndrome-Causing SBDS c.258+2T>C Mutation by RNA Therapeutics and Base/Prime Editing
Source: Int J Mol Sci. 2023 Feb 16;24(4):4024. doi: 10.3390/ijms24044024 (PMC9962285; doi:10.3390/ijms24044024)
Supplement: Supplementary file 1 [file ijms-24-04024-s001.zip › ijms-2094187-supplementary.pdf]

Table S1. List of primers exploited in the study.

Name	Sequence 5′-3′
**Primers for creation of PTM molecules**	
BD1 BamHI F	GGATCCTGTTGTTGCCATCTCGTATT
BD1 HindIII R	AAGCTTCAGAACTTCATCGAGGTCTTT
BD2 BamHI F	GGATCCACAGTGAAATTCCATCTAGGG
BD2 HindIII R	AAGCTTCGGCAAGACACAACAAATC
BD3 BamHI F	GGATCCGGCGCATGCCTGTAATC
BD3 HindIII R	AAGCTTCGATCTCGGGTCACTGTAA
BD1i HindIII F	AAGCTTTGTTGTTGCCATCTCGTATT
BD1i BamHI R	GGATCCCAGAACTTCATCGAGGTCTTT
BD2i HindIII F	AAGCTTACAGTGAAATTCCATCTAGGG
BD2i BamHI R	GGATCCCGGCAAGACACAACAAATC
BD3i HindIII F	AAGCTTGGCGCATGCCTGTAATC
BD3i BamHI R	GGATCCCGATCTCGGGTCACTGTAA
Donor F	GTAAGTATCAAGGTTACAAGACAGG
Donor R	GGATCCAATGAATTGAGAAACC
Intron F	ACTAGTAACGGCCGCCAGTG
Intron R	CCTCGACCGCCCTGAGGGAGGGAAAATAGACCAATAG
GFPt F	CCCTCCCTCAGGGCGGTCGAGGACAAACTC
GFPt R	GGTGACACTATAGAATAGGGCCCTC
**Primers for creation of g/pegRNA**	
sgBE F	ACACCGGCAGGCGGGTAACAGCTGCG
sgBE R	AAAACGCAGCTGTTACCCGCCTGCCG
peg1 sg F	CACCGGCTGCAGCTGTTACCCGCCGTTTTAGA
peg1 sg R	TAGCTCTAAAACGGCGGGTAACAGCTGCAGCC
peg1 ext. F	GTGCTCTGTAAGCAGGTGGGTAACAGCTG
peg1 ext. R	AAAACAGCTGTTACCCACCTGCTTACAGA
peg2 sg F	CACCGTGAAATCTGTAAGCAGGCGGTTTTAGA
peg2 sg R	TAGCTCTAAAACCGCCTGCTTACAGATTTCAC
peg2 ext. F	GTGCAGCTGTTACCCACCTGCTTACAGATTT
peg2 ext. R	AAAAAAATCTGTAAGCAGGTGGGTAACAGCT
peg3 sg F	CACCGCTGAAATCTGTAAGCAGGCGTTTTAGA
peg3 sg R	TAGCTCTAAAACGCCTGCTTACAGATTTCAGC
peg3 ext. F	GTGCAGCTGTTACCCACCTGCTTACAGATTTC
peg3 ext. R	AAAAGAAATCTGTAAGCAGGTGGGTAACAGCT
scaff F	GCTAGAAATAGCAAGTTAAAATAAGGCTAGTCCGTTATCAACTTGAAAAA GTGGCACCGAGTCG
scaff R	GCACCGACTCGGTGCCACTTTTTCAAGTTGATAACGGACTAGCCTTATTTT AACTTGCTATTTC
**Primers for cloning and mRNA studies**	
SBDS IVS1 F NdeI	AAAAAACATATGCAGTTTAAAGTGCGTAGTGTCTTC
SBDS IVS2 R NdeI	AAAAAACATATGAAGCTGAGGCAAGAGAATCG
Alfa2-3 globin F	CAACTTCAAGCTCCTAAGCCACTGC
BRA2 Rev	GTCACCAGGAAGTTGGTTAAATCA
eGFP R	GTTTACGTCGCCGTCCAGC
SBDS 1F	AATCGCCTGCTACAAAAACAAG
qSBDS ex2F	TGACCAAACTGAAATCTGTAAGCAG
qSBDS ex3R	AGTGGATGTCCTTCATGGCTC

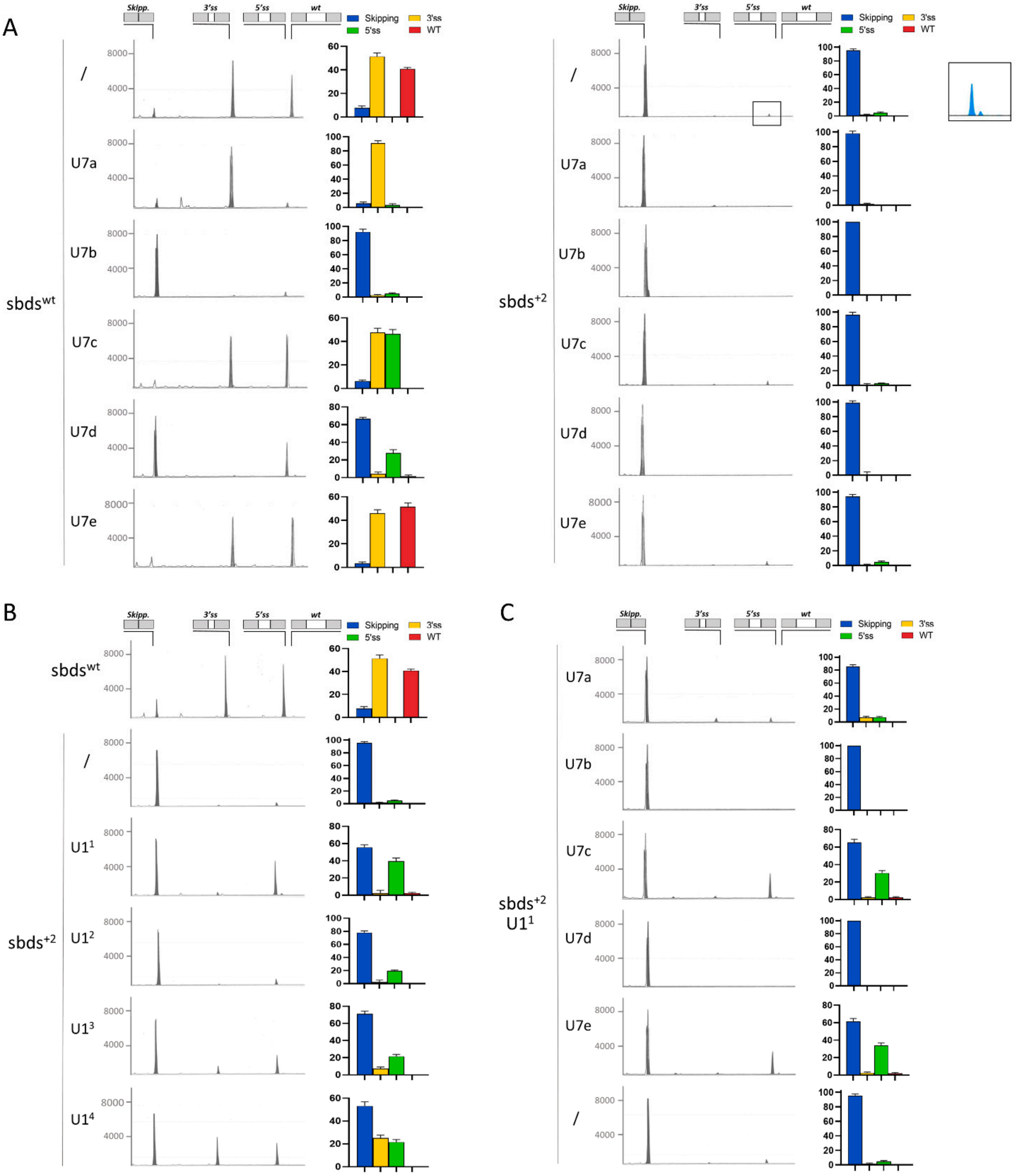

Figure S1. Characterization of splicing elements within SBDS exon 2 and evaluation of engineered U1snRNAs and U7snRNA as possible therapeutic tools by denaturing capillary electrophoresis of labelled transcripts. The schematic representation of transcripts is reported on top. Quantification of transcripts is reported on right. Results are presented as mean ± SD of three independent experiments.

```
SBDS            ATGTCGATCTTCACCCCCACCAACCAGATCCGCCTAACCAATGTGGCCGTGGTACGGATG
SBDSP           ATGTCGATCTTCACCCCCACCAACCAGATCCGCCTAACCAATGTGGCCGTGGTACGGATG
                ************************************************************

SBDS            AAGCGTGCCGGGAAGCGCTTCGAAATCGCCTGCTACAAAAACAAGGTCGTCGGCTGGCGG
SBDSP           AAGCGCGCCAGGAAGCGCTTCGAAATCGCCTGCTACAAAAACAAGGTCGTCGGCTGGCGG
                ***** *** **************************************************

SBDS            AGCGGCGT
SBDSP           AGCGGCTT
                ****** *
```

Figure S2. Multiple sequence alignment of DNA of SBDS and SBDSP exon 1.

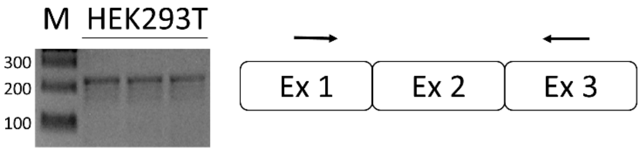

Figure S3. Evaluation of SBDS expression in HEK293T cells. Scheme reports the expected transcript and primers, indicated by arrow, exploited.

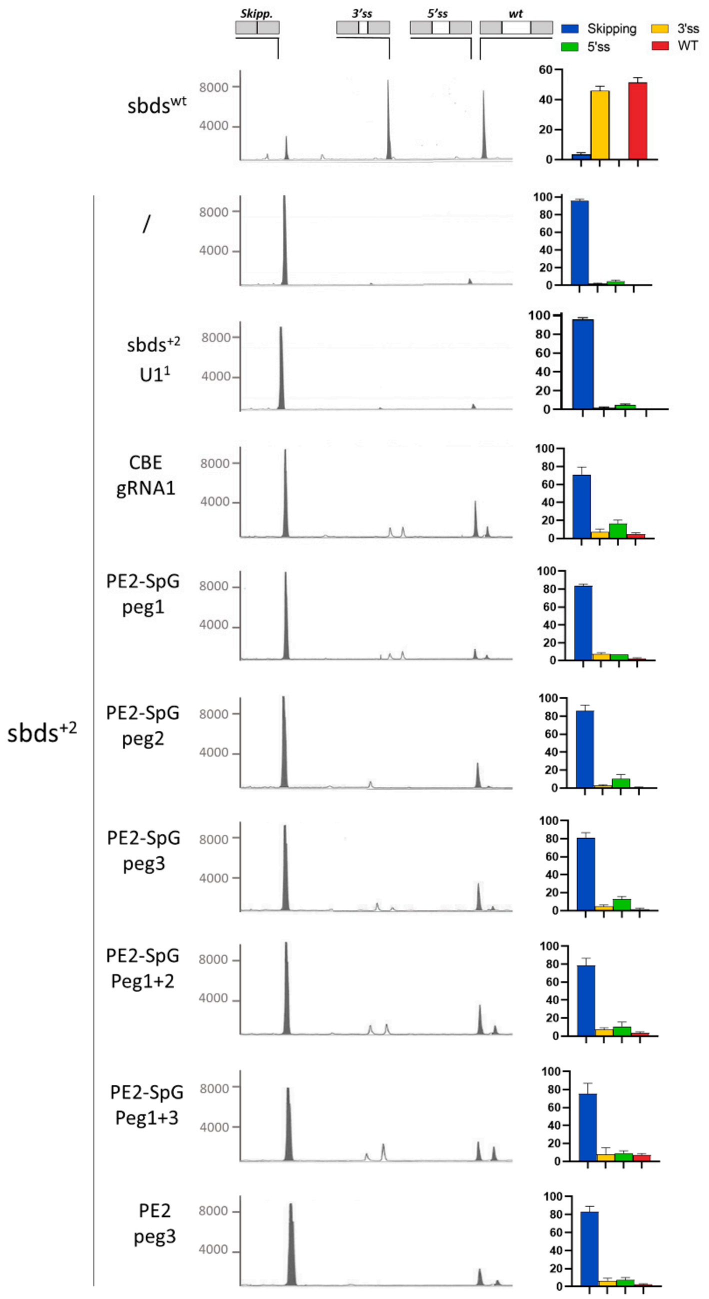

Figure S4. Evaluation by denaturing capillary electrophoresis of labelled transcripts of base and prime editing tools as correction approaches**.** The schematic representation of transcripts is reported on top. Quantification of transcripts is reported on right. Results are presented as mean ± SD of three independent experiments.

A

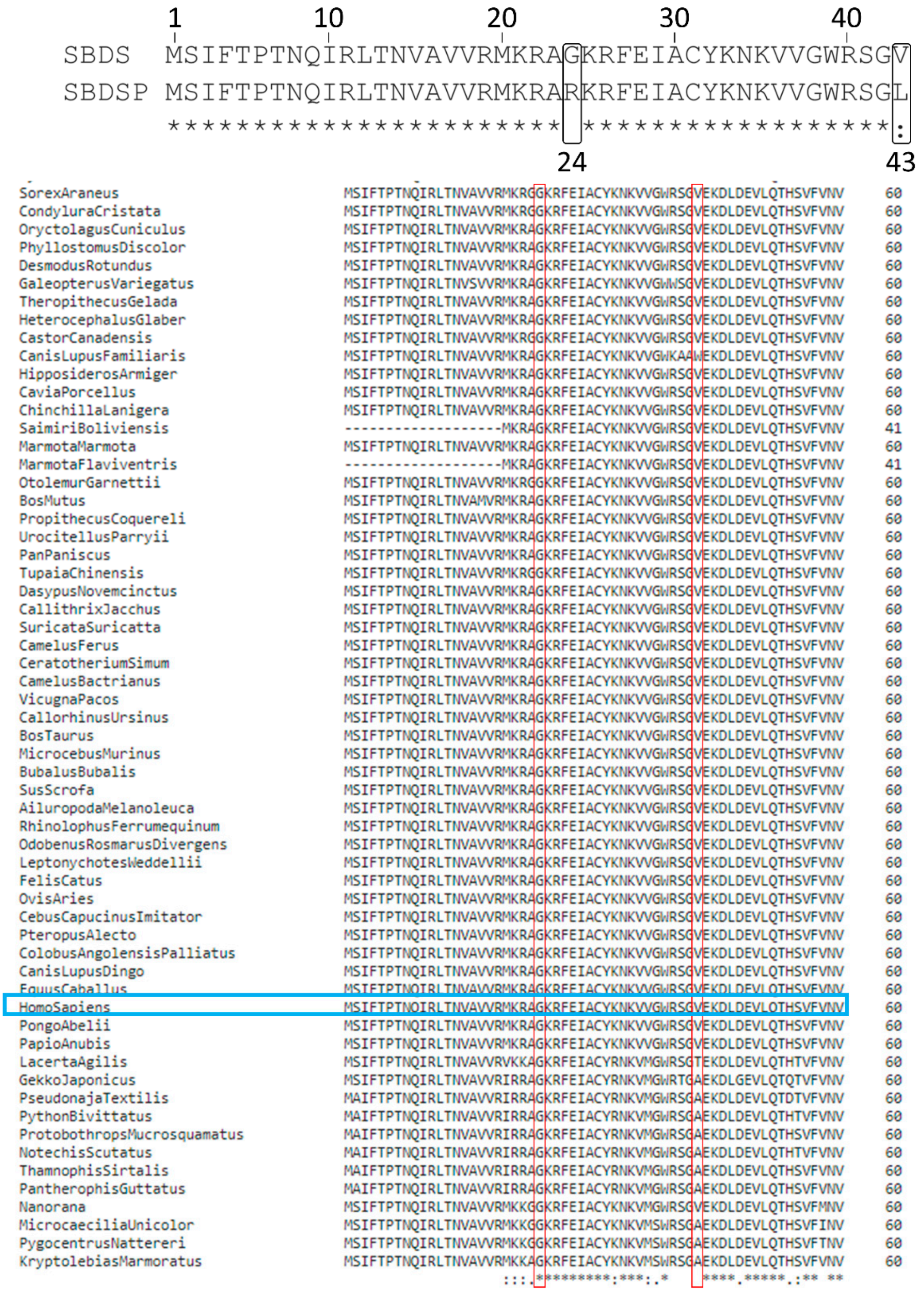
1 10 20 30 40
SBDS MSIFTPTNQIRLTNVAVVRMKRAGKRFEIACYKNKVVGWRSGV
SBDSP MSIFTPTNQIRLTNVAVVRMKRARKRFEIACYKNKVVGWRSGL
*********************** ******************:
24 43
SorexAraneus MSIFTPTNQIRLTNVAVVRMKRGGKRFEIACYKNKVVGWRSGVEKDLDEVLQTHSVFVNV 60
CondyluraCristata MSIFTPTNQIRLTNVAVVRMKRGGKRFEIACYKNKVVGWRSGVEKDLDEVLQTHSVFVNV 60
OryctolagusCuniculus MSIFTPTNQIRLTNVAVVRMKRAGKRFEIACYKNKVVGWRSGVEKDLDEVLQTHSVFVNV 60
PhyllostomusDiscolor MSIFTPTNQIRLTNVAVVRMKRAGKRFEIACYKNKVVGWRSGVEKDLDEVLQTHSVFVNV 60
DesmodusRotundus MSIFTPTNQIRLTNVAVVRMKRAGKRFEIACYKNKVVGWRSGVEKDLDEVLQTHSVFVNV 60
GaleopterusVariegatus MSIFTPTNQIRLTNVSVVRMKRAGKRFEIACYKNKVVGWWSGVEKDLDEVLQTHSVFVNV 60
TheropithecusGelada MSIFTPTNQIRLTNVAVVRMKRAGKRFEIACYKNKVVGWRSGVEKDLDEVLQTHSVFVNV 60
HeterocephalusGlaber MSIFTPTNQIRLTNVAVVRMKRAGKRFEIACYKNKVVGWRSGVEKDLDEVLQTHSVFVNV 60
CastorCanadensis MSIFTPTNQIRLTNVAVVRMKRGGKRFEIACYKNKVVGWRSGVEKDLDEVLQTHSVFVNV 60
CanisLupusFamiliaris MSIFTPTNQIRLTNVAVVRMKRAGKRFEIACYKNKVVGWKAAWEKDLDEVLQTHSVFVNV 60
HipposiderosArmiger MSIFTPTNQIRLTNVAVVRMKRAGKRFEIACYKNKVVGWRSGVEKDLDEVLQTHSVFVNV 60
CaviaPorcellus MSIFTPTNQIRLTNVAVVRMKRAGKRFEIACYKNKVVGWRSGVEKDLDEVLQTHSVFVNV 60
ChinchillaLanigera MSIFTPTNQIRLTNVAVVRMKRAGKRFEIACYKNKVVGWRSGVEKDLDEVLQTHSVFVNV 60
SaimiriBoliviensis -------------------MKRAGKRFEIACYKNKVVGWRSGVEKDLDEVLQTHSVFVNV 41
MarmotaMarmota MSIFTPTNQIRLTNVAVVRMKRAGKRFEIACYKNKVVGWRSGVEKDLDEVLQTHSVFVNV 60
MarmotaFlaviventris -------------------MKRAGKRFEIACYKNKVVGWRSGVEKDLDEVLQTHSVFVNV 41
OtolemurGarnettii MSIFTPTNQIRLTNVAVVRMKRGGKRFEIACYKNKVVGWRSGVEKDLDEVLQTHSVFVNV 60
BosMutus MSIFTPTNQIRLTNVAMVRMKRAGKRFEIACYKNKVVGWRSGVEKDLDEVLQTHSVFVNV 60
PropithecusCoquereli MSIFTPTNQIRLTNVAVVRMKRAGKRFEIACYKNKVVGWRSGVEKDLDEVLQTHSVFVNV 60
UrocitellusParryii MSIFTPTNQIRLTNVAVVRMKRAGKRFEIACYKNKVVGWRSGVEKDLDEVLQTHSVFVNV 60
PanPaniscus MSIFTPTNQIRLTNVAVVRMKRAGKRFEIACYKNKVVGWRSGVEKDLDEVLQTHSVFVNV 60
TupaiaChinensis MSIFTPTNQIRLTNVAVVRMKRGGKRFEIACYKNKVVGWRSGVEKDLDEVLQTHSVFVNV 60
DasypusNovemcinctus MSIFTPTNQIRLTNVAVVRMKRAGKRFEIACYKNKVVGWRSGVEKDLDEVLQTHSVFVNV 60
CallithrixJacchus MSIFTPTNQIRLTNVAVVRMKRAGKRFEIACYKNKVVGWRSGVEKDLDEVLQTHSVFVNV 60
SuricataSuricatta MSIFTPTNQIRLTNVAVVRMKRAGKRFEIACYKNKVVGWRSGVEKDLDEVLQTHSVFVNV 60
CamelusFerus MSIFTPTNQIRLTNVAVVRMKRAGKRFEIACYKNKVVGWRSGVEKDLDEVLQTHSVFVNV 60
CeratotheriumSimum MSIFTPTNQIRLTNVAVVRMKRAGKRFEIACYKNKVVGWRSGVEKDLDEVLQTHSVFVNV 60
CamelusBactrianus MSIFTPTNQIRLTNVAVVRMKRAGKRFEIACYKNKVVGWRSGVEKDLDEVLQTHSVFVNV 60
VicugnaPacos MSIFTPTNQIRLTNVAVVRMKRAGKRFEIACYKNKVVGWRSGVEKDLDEVLQTHSVFVNV 60
CallorhinusUrsinus MSIFTPTNQIRLTNVAVVRMKRAGKRFEIACYKNKVVGWRSGVEKDLDEVLQTHSVFVNV 60
BosTaurus MSIFTPTNQIRLTNVAVVRMKRAGKRFEIACYKNKVVGWRSGVEKDLDEVLQTHSVFVNV 60
MicrocebusMurinus MSIFTPTNQIRLTNVAVVRMKRAGKRFEIACYKNKVVGWRSGVEKDLDEVLQTHSVFVNV 60
BubalusBubalis MSIFTPTNQIRLTNVAVVRMKRAGKRFEIACYKNKVVGWRSGVEKDLDEVLQTHSVFVNV 60
SusScrofa MSIFTPTNQIRLTNVAVVRMKRAGKRFEIACYKNKVVGWRSGVEKDLDEVLQTHSVFVNV 60
AiluropodaMelanoleuca MSIFTPTNQIRLTNVAVVRMKRAGKRFEIACYKNKVVGWRSGVEKDLDEVLQTHSVFVNV 60
RhinolophusFerrumequinum MSIFTPTNQIRLTNVAVVRMKRAGKRFEIACYKNKVVGWRSGVEKDLDEVLQTHSVFVNV 60
OdobenusRosmarusDivergens MSIFTPTNQIRLTNVAVVRMKRAGKRFEIACYKNKVVGWRSGVEKDLDEVLQTHSVFVNV 60
LeptonychotesWeddellii MSIFTPTNQIRLTNVAVVRMKRAGKRFEIACYKNKVVGWRSGVEKDLDEVLQTHSVFVNV 60
FelisCatus MSIFTPTNQIRLTNVAVVRMKRAGKRFEIACYKNKVVGWRSGVEKDLDEVLQTHSVFVNV 60
OvisAries MSIFTPTNQIRLTNVAVVRMKRAGKRFEIACYKNKVVGWRSGVEKDLDEVLQTHSVFVNV 60
CebusCapucinusImitator MSIFTPTNQIRLTNVAVVRMKRAGKRFEIACYKNKVVGWRSGVEKDLDEVLQTHSVFVNV 60
PteropusAlecto MSIFTPTNQIRLTNVAVVRMKRAGKRFEIACYKNKVVGWRSGVEKDLDEVLQTHSVFVNV 60
ColobusAngolensisPalliatus MSIFTPTNQIRLTNVAVVRMKRAGKRFEIACYKNKVVGWRSGVEKDLDEVLQTHSVFVNV 60
CanisLupusDingo MSIFTPTNQIRLTNVAVVRMKRAGKRFEIACYKNKVVGWRSGVEKDLDEVLQTHSVFVNV 60
EquusCaballus MSIFTPTNQIRLTNVAVVRMKRAGKRFEIACYKNKVVGWRSGVEKDLDEVLQTHSVFVNV 60
HomoSapiens MSIFTPTNQIRLTNVAVVRMKRAGKRFEIACYKNKVVGWRSGVEKDLDEVLQTHSVFVNV 60
PongoAbelii MSIFTPTNQIRLTNVAVVRMKRAGKRFEIACYKNKVVGWRSGVEKDLDEVLQTHSVFVNV 60
PapioAnubis MSIFTPTNQIRLTNVAVVRMKRAGKRFEIACYKNKVVGWRSGVEKDLDEVLQTHSVFVNV 60
LacertaAgilis MSIFTPTNQIRLTNVAVVRVKKAGKRFEIACYRNKVMGWRSGTEKDLDEVLQTHTVFVNV 60
GekkoJaponicus MSIFTPTNQIRLTNVAVVRIRRAGKRFEIACYRNKVMGWRTGAEKDLGEVLQTQTVFVNV 60
PseudonajaTextilis MAIFTPTNQIRLTNVAVVRIRRAGKRFEIACYRNKVMGWRSGAEKDLDEVLQTDTVFVNV 60
PythonBivittatus MAIFTPTNQIRLTNVAVVRIRRAGKRFEIACYRNKVMGWRSGAEKDLDEVLQTHTVFVNV 60
ProtobothropsMucrosquamatus MAIFTPTNQIRLTNVAVVRIRRAGKRFEIACYRNKVMGWRSGAEKDLDEVLQTHSVFVNV 60
NotechisScutatus MAIFTPTNQIRLTNVAVVRIRRAGKRFEIACYRNKVMGWRSGAEKDLDEVLQTHTVFVNV 60
ThamnophisSirtalis MAIFTPTNQIRLTNVAVVRIRRAGKRFEIACYRNKVMGWRSGAEKDLDEVLQTHSVFVNV 60
PantherophisGuttatus MAIFTPTNQIRLTNVAVVRIRRAGKRFEIACYRNKVMGWRSGAEKDLDEVLQTHSVFVNV 60
Nanorana MSIFTPTNQIRLTNVAVVRMKKGGKRFEIACYKNKVMGWRSGVEKDLDEVLQTHSVFMNV 60
MicrocaeciliaUnicolor MSIFTPTNQIRLTNVAVVRMKKGGKRFEIACYKNKVMSWRSGAEKDLDEVLQTHSVFINV 60
PygocentrusNattereri MSIFTPTNQIRLTNVAVVRMKKGGKRFEIACYKNKVMSWRSGAEKDLDEVLQTHSVFTNV 60
KryptolebiasMarmoratus MSIFTPTNQIRLTNVAVVRMKKAGKRFEIACYKNKVMSWRSGAEKDLDEVLQTHSVFVNV 60
::::.*********:***:.* ****.*****.:** **

B

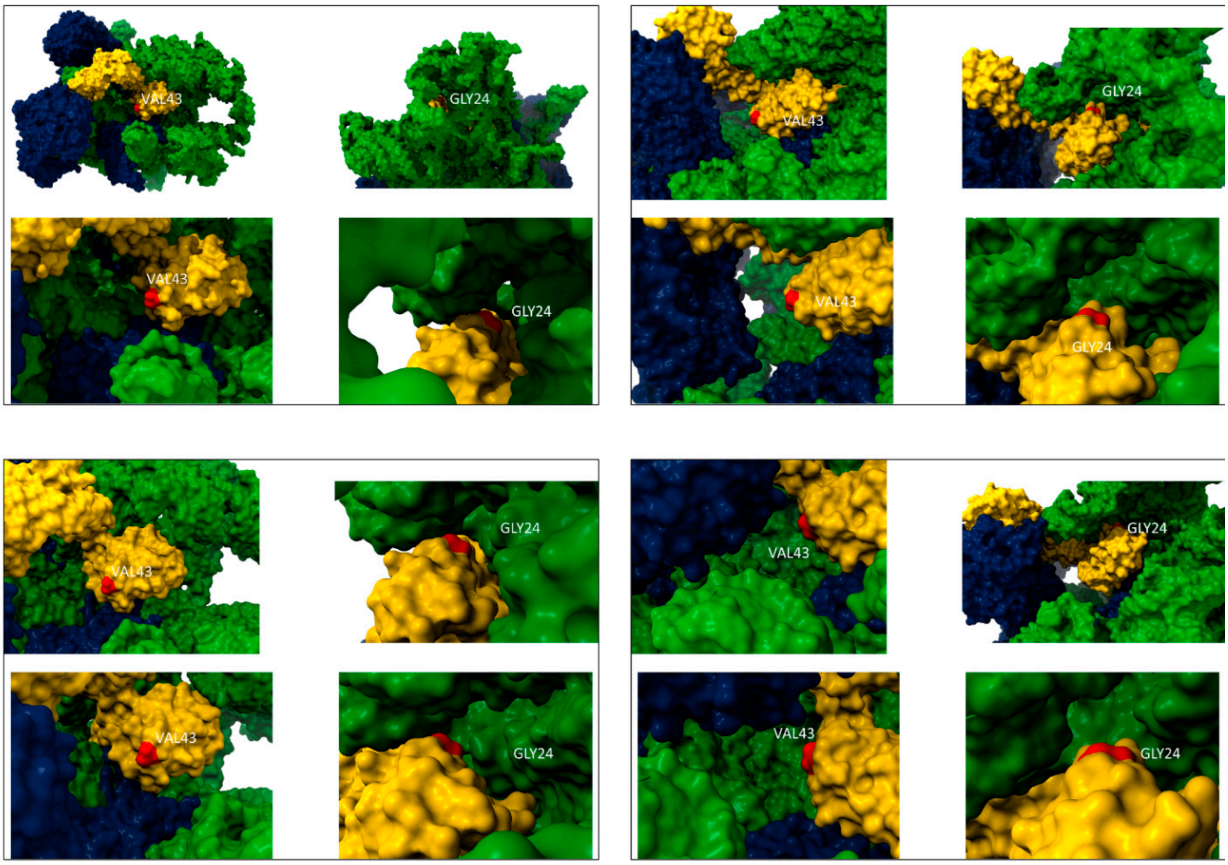

Figure S5. Conservation of SBDS exon 1 among species and Analysis of 3D structures **(A)** Multiple sequence alignment of protein coded by *SBDS* and *SBDSP* exon 1 among species. The human sequence is indicated by the blue rectangle. Direct comparison between *SBDS* and *SBDSP* is shown on top; **(B)** Inspection of molecular structures during 60S ribosomal subunit formation (PDBs: 5an9, 5anb, 5anc and 6qkl). SBDS, rRNA and other proteins of the 60S ribosomal subunit are indicated in yellow, green and blue, respectively. SBDS G24 is located in the interface with rRNA and thus the p.G24R variant could alter rRNA contacts that are critical for 60S binding and the stabilization of functionally important conformational states [45]. On the other hand, SBDS position 43 could be more tolerant to aminoacid changes, since located within a bucket without any interaction with other SBDS or 60S ribosomal subunits residues.

Supplementary References:

45. Weis, F.; Giudice, E.; Churcher, M.; Jin, L.; Hilcenko, C.; Wong, C.C.; Traynor, D.; Kay, R.R.; Warren, A.J. Mechanism of EIF6 Release from the Nascent 60S Ribosomal Subunit. *Nature Structural and Molecular Biology* **2015**, *22*, 914–919, doi:10.1038/nsmb.3112.
